# Multi-generational fidelity, ecological and social determinants of roosting in a cooperatively breeding bird (*Argya squamiceps*)

**DOI:** 10.1098/rsos.251180

**Published:** 2025-11-26

**Authors:** Yitzchak Ben Mocha, Itamar Ring, Sophie Scemama de Gialluly, Oded Keynan

**Affiliations:** ^1^ Centre for the Advanced Study of Collective Behaviour, University of Konstanz, Konstanz, Germany; ^2^ Zukunftskolleg, University of Konstanz, Konstanz, Germany; ^3^ Dead Sea and Arava Science Center, Neve Zohar, Israel; ^4^ Negev-Eilat Campus, Ben-Gurion University of the Negev, Eilat, Israel

**Keywords:** Arabian babbler, sleep, group size, acacia, conservation, *Plicosepalus acacia*, animal traditions

## Abstract

Sleep is an important but overlooked component of animal behaviour, especially its social and conservation facets. Here, we use 15 years of data to comprehensively describe the roosting behaviour of cooperatively breeding birds and test hypotheses about its ecological and social determinants. We show that wild Arabian babbler groups in the Arava Desert of Israel preferred roosting in live plants with dense canopies (mostly *Acacia* tree spp. and reed clusters). Roosting sites were located in the inner areas of territories regardless of territorial conflicts. Groups almost always roosted in intimate huddles but tended to separate into sub-groups that roost in nearby trees as group size increased. Despite the abundance of suitable sites for roosting, each group only used an average of 2.4 main roosting sites within its territory. Social groups thus exhibited strong, non-random fidelity to specific roosting sites that extended over ≥4 group generations and ≥15 years. To the best of our knowledge, this is the longest roosting site fidelity shown for cooperatively breeding birds and mammals. This study stresses the importance of conserving roosting sites of species with strong site fidelity and lays the foundations for advanced sleep research in a highly cooperative species.

## 1. Introduction

Sleep is a fundamental physiological need in birds and mammals. Without quantitatively and qualitatively sufficient sleep, physiological and cognitive abilities may be impaired to a life-threatening level [1]. Despite the importance of sleep, field data are often collected when wild animals are active. This bias has led to the diverse ecological and social factors supporting sleep being relatively overlooked in animal behaviour research [2–4].

Difficulties in studying wild animals when they seek elusiveness during the vulnerable state of sleep [5,6] result in two main knowledge gaps. First, little is known about the roosting behaviour of many species. Specifically, which ecological and social criteria are used to select roosting sites? Protection from predators [7,8] and/or from cold and wet climates [9–11] is hypothesized to be an important ecological criterion in birds and mammals. Proximity to the territory’s border is important in Spix’s moustached tamarins *Saguinus mystax* [7] and green woodhoopoes *Phoeniculus purpureus,* which roost along their territorial borders following inter-group conflicts [12]. In addition, group members in cooperatively breeding species tend to spend most of their daytime in proximity [13,14], yet little is known about their social cohesion during sleep. While groups splitting to roost in different sites were observed in several species, the frequency of splitting and whether it occurs owing to insufficient space in a single site (e.g. cotton-top tamarins *Saguinus oedipus* [15]*,* green woodhoopoes [16]), loose social relationships [17] and/or other reasons [11] remain to be determined per species.

A second major knowledge gap is the unclarity about whether roosting sites are used opportunistically [7,18] or specific sites are being used regularly. Further, if specific sites are being used regularly, is it because of their special merits, lack of suitable alternatives [12,19] or ‘social traditions’ [20]? Here, the paucity of data is largely owing to: (i) short-term studies that cannot assess long-term roosting patterns; and (ii) observations of unhabituated wild animals make it difficult to determine whether frequent changes in roosting sites occur naturally or are attempts to avoid interruption by human observers [21,22]. Fidelity to specific roosting sites will require conserving these sites [18,22].

**Figure 1.**
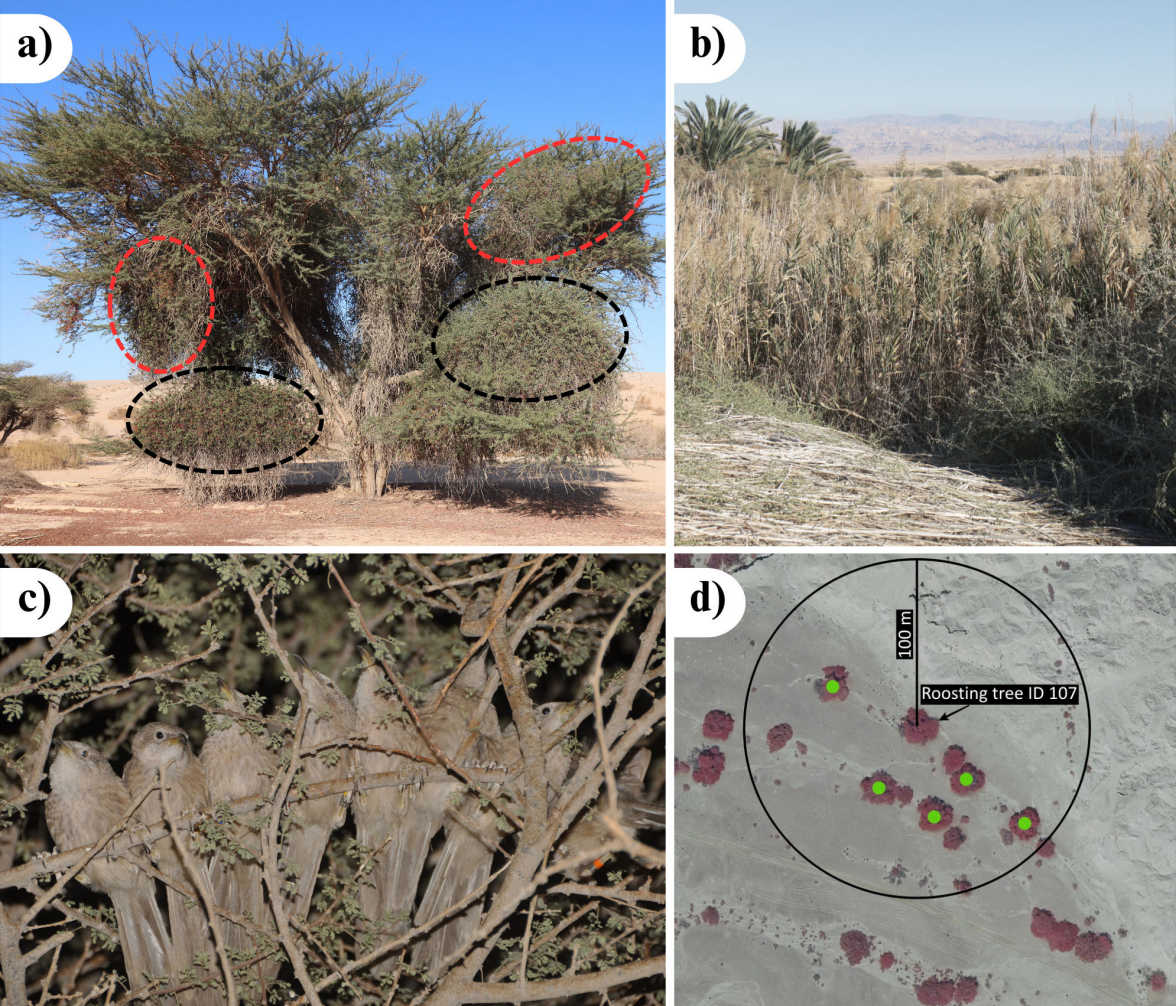
(a) An *Acacia raddiana* roosting tree with a relatively poor canopy cover, but with an extensive growth of *Plicosepalus acacia* clusters in which the MTE group has had roosts since 2011 (non-roosting/roosting *Plicosepalus acacia* clusters are circled in black/red, respectively). (b) A roosting cluster of common reeds (*Phragmites australis*). Photos (a) and (b) by I.R. (c) A social group of Arabian babblers in a typical roosting huddle. Photo: Y.B.M. (d) A colour-infrared satellite image of group-area combination HOR_107 (adapted from www.govmap.gov.il). Tree 107 was used for roosting by the HOR group for 18 out of 20 observation nights during five months. Throughout this period, we have not observed any Arabian babbler group roosting in the other five similar-sized acacia trees within a 100 m radius (marked in green), nor in the other trees in the image.

Here, we advance knowledge in the above domains by studying the roosting behaviour of a cooperatively breeding bird: the Arabian babbler (*Argya squamiceps*). Arabian babblers inhabit the extreme deserts of the Arabian and Sinai Peninsulas, Palestine and Israel [23]. Their habitat is characterized by high day temperatures that often last during the night-time (annual mean: 24.4°C; summer mean: 31.8°C [24]). The habitat is hyper-arid, and there are only a few rainy days a year (mean annual rainfall: 48.2 mm [24]). These medium-sized songbirds (adult body mass: 66—77 g [25]) have a mean lifespan of 3.2 years (O Keynan, 1978-2013 unpublished data). They live in stable social groups of 2–20 birds of both sexes and diverse ages [26]. Since all group members care for the young (e.g. feeding [27] and transporting fledglings between locations [28]), the species is considered a cooperative breeder [29,30]. Each group defends a permanent territory year-round, in which group members move cohesively [13]. Arabian babblers are diurnal birds that strictly avoid activity during the dark [24]. Around the time of evening civil twilight [24], a group would retreat to a roosting site (i.e. figure 1a,b) where the birds form a linear huddle along an inner branch ([31]; figure 1c).

The specific goals of the study are threefold. First, method validation. We verified that our observations do not alter natural roosting behaviour [7,32] in two complementary ways. We compared the frequency of: (i) roosting site change during the night-time; and (ii) change of roosting site across nights, both under an experimental condition (i.e. observation by a human) versus a control condition (i.e. automatic audio recording). If human presence during settling into sleep and/or waking up considerably disturbs the birds, we predicted they would change roosting sites during night-time and/or across nights more often under human observations.

Second, we describe key ecological and social factors shaping Arabian babblers’ roosting behaviour, including testing hypotheses about factors thought to influence roosting in cooperatively breeding birds and mammals: (i) whether canopy density and width are important criteria for roosting sites [33,34]; (ii) whether the location of roosting sites is affected by territorial borders and/or conflicts [7,12]; and (iii) whether social groups split to roost in different sites (i.e. different trees or reed clusters) because of insufficient space at a single roosting site and/or because the group is too large [15–17].

Third, we used 15 years of data to test Zahavi’s [20] hypothesis that Arabian babblers exhibit strong fidelity to specific roosting trees. Specifically, we (i) tested whether groups roost opportunistically in different sites or use specific roosting sites significantly more often than others. Following confirmation of strong roosting site fidelity, we tested (ii) whether this roosting site fidelity is owing to a lack of alternative suitable roosting sites [16], and (iii) across how many generations of the social group this site fidelity persists.

## 2. Methods

### 2.1. Behavioural observations

The study was conducted in a 62 km^2^ area including the Shezaf nature reserve and its surroundings in Israel (30.73° N, 35.27° E). Continuous monitoring, individual marking and habituation to human researchers of the Arabian babbler population since 1971 provide comprehensive knowledge of the life history of individuals and groups [35,36]. We conducted behavioural observations throughout November 2011–July 2012, February 2014–June 2014 and January 2020–July 2025. In the evenings, we followed the focal group from 1–2 h before dusk until it reached its roosting site. To ensure the birds did not change roosting site later in the evening, we recorded the location of the roosting site after all group members stayed in a roosting huddle for 2 min or disappeared into dense vegetation (figure 1c) and remained quiet for at least 2 min [2,37]. In the mornings, we waited at known roosting sites before civil twilight [24] and recorded whether the group emerged from the trees/reeds. The group ID, Global Positioning System (GPS) coordinates of the roosting site and timestamps were recorded on a smartphone using CyberTracker [38] software with an accuracy of 3–40 m.

### 2.2. Data filtering, preparation and analysis

Roosting of ‘floater’ birds (i.e. a single bird transiting between social groups, *n* = 3 floaters, three nights) was excluded from all analyses. Trees inside plantations (*n* = 4 trees, four groups) were only used for the descriptive characteristics of roosting sites (e.g. table 1). Analyses evaluating the usage frequency of roosting sites were limited to data from 22 extensively studied groups (i.e. >10 observation nights, 14–124 nights per group).

**Table 1.** Plant species used for roosting (*n* = 82).

English name	Latin name	number of roosting sites	percentage (%)
umbrella thorn acacia	*Acacia tortilis*	36	43.9
twisted acacia	*Acacia raddiana*	31	37.8
common reeds (cluster)	*Phragmites australis*	5	6.1
Christ’s thorn jujube	*Ziziphus spina-christi*	2	2.4
athel pine	*Tamarix aphylla*	1	1.2
date palm	*Phoenix dactylifera*	2	2.4
mango plantation		3	3.7
lemon plantation		1	1.2
hedge		1	1.2

R (v. 4.5.1 [39]) was used for data preparation and analyses. Generalized linear mixed models (hereafter GLMM) were fitted with the glmmTMB function (glmmTMB package, v. 1.1.10 [40]), using a binomial error structure and logit link function [41,42]. The normality of residuals and the absence of outliers, under- and over-dispersion were examined using the DHARMa package (v. 0.4.6 [43]). All tests were two-tailed, and the significance level was set to 0.05. All but one GLMM had a single fixed effect, and in these models, the statistical significance of the fixed effect was tested by comparing the full model with its ‘null’ version that contained only the intercept and random effects using a likelihood ratio test (R function ‘ANOVA’ with argument test ‘*x*^2^’ [44]). See below for the model with multiple fixed effects.

### 2.3. Method validation

#### 2.3.1. Do trained human observers alter roosting site usage?

The vocalizations of Arabian babblers while organizing and breaking the roosting huddle [20] enabled us to compare roosting behaviour under human observation versus under automatic audio recording. A trained researcher observed eight Arabian babbler groups for five to six consecutive nights each (one group was observed only in the mornings, two groups were observed only in the evenings and five groups were observed in the evenings and the following mornings of each night). On the last day, an AudioMoth (v. 1.2.0 [45]) was installed 1–3 m away from the roosting branch of 2–5 known roosting trees of each group. AudioMoths were set to record (sampling rate of 96 kHz) continuously from 1 h before the typical time Arabian babblers set to roost until 1 h after the typical time they wake up (i.e. evening/morning civil twilight, respectively [24]). Recordings were made during each night until battery failure (6–34 consecutive nights per group).

The human effect was examined in two complementary ways. First, we compared the rate of roosting site change during night-time between the control (i.e. automatic audio recording) and experimental (i.e. human observer) conditions. A group was considered not to change site if a researcher observed it or if Arabian babbler vocalizations were recorded in the same tree during the evening and subsequent morning. Since Arabian babblers are highly territorial and we have never observed a group roosting within another group’s territory, we assumed that the recorded vocalizations belong to the group owning this territory. For this ‘within-night’ GLMM, the response variable was whether the group changed the roosting site during night-time (yes/no). The experimental condition (human observer/AudioMoth) was set as a fixed effect, and group ID and tree ID as random effects. The sample size was 23 groups, 40 trees, 118 nights of AudioMoth recordings and 132 nights under human observation (including nights that were not part of the continuous experiment).

Second, we compared the rate of changing roosting sites across nights between the two experimental conditions. A group was considered roosting on a tree during a specific night if it was observed by a researcher or recorded by an AudioMoth during the evening and/or morning of the focal night. The response variable of this ‘between nights’ GLMM was whether the group roosted in its main roosting site during this period (yes/no). The experimental condition (human observer/AudioMoth) was set as a fixed effect, and group ID as a random effect (since each group roosted on the same tree during almost all nights, group ID and tree ID were statistically indistinguishable from one another). The sample size was eight groups, 121 nights of AudioMoth recording and 42 observation nights by a human observer.

### 2.4. Ecological and social characteristics of roosting behaviour

#### 2.4.1. What characteristics are important for roosting sites?

To examine the criteria used to select a roosting site over nearby alternatives, the coordinates of all roosting sites were uploaded to the Google Earth Pro software. The immediate surroundings of each site (i.e. a 100 m radius circle) were then marked (figure 1d). Each circle was linked to the group that roosted on the focal site and was given a group-area combination ID. Two types of group-area combinations were created: (i) for roosting sites whose 100 m radius did not overlap with other roosting sites of the group, the group-area combination was this single 100 m radius (*n* = 65 group-area combinations); and (ii) for roosting sites whose 100 m radius overlapped with other roosting sites used by the same group, the group-area combination was the combined 100 m radii of the overlapping roosting sites (*n* = 16 group-area combinations).

Next, we counted the number of potential sites within each group-area combination. In group-area combinations with roosting trees, we counted the number of trees (and reed clusters) with canopy size (area) similar to or larger than the focal roosting tree. This is a conservative approach since all group-area combinations included additional trees smaller than the focal roosting tree. In group-area combinations with roosting reed clusters, we counted the number of reed clusters and trees with a canopy size greater than the average roosting tree. We did so because reed clusters were considerably larger than trees and always had large trees near them.

Trees of similar size to the roosting tree were identified in three sequential steps. We: (i) analysed a recent, high-resolution colour-infrared satellite image from www.govmap.gov.il (figure 1d); (ii) verified that the same trees were present in satellite images taken within 0–4 years of the collection of behavioural data (using the historical imagery in Google Earth Pro and www.govmap.gov.il); and subsequently (iii) surveyed each potential roosting site in the field.

During our field survey, we recorded for each site: (i) the plant species; (ii) whether the plant was alive or dead; (iii) a three-level categorical index of canopy density (1 = the main branches of the tree are clearly visible, 2 = the main branches of the tree are somehow visible, 3 = at least one main branch of the tree is hidden by very dense foliage (electronic supplementary material, figure S1) and/or extensive growth of vines (e.g. *Plicosepalus acacia*; figure 1a); and took a photo to measure (iv) canopy width and (v) tree height. Each photo included a 1 m long measuring rod (50 cm coloured in red and 50 cm coloured in white) vertically hung on the tree. ImageJ software [46] was used to measure the width between the two canopy edges and tree height from ground to the canopy’s upper edge, using the rod as a reference. In both cases, we considered the edges of the canopy as the most extreme horizontal branches big enough for two Arabian babblers to stand on.

We ran multiple GLMMs with all the possible combinations of fixed effects (without interaction) and chose the model with the lowest score of Bayesian Information Criterion (BIC). In all model combinations, the response variable was whether the site (a tree or a reed cluster) had been used for roosting at least once (yes/no), and a log of the total number of sites within each area-group combination was used as an offset to account for the variability in the number of sites across group-area combinations. Four biologically meaningful fixed effects were examined: (i) the plant species; (ii) canopy density (coded as a factor); (iii) tree height (in cm); and (iv) canopy width (in cm). This model included only the 220 sites (out of 560) for which we had canopy and height measurements and those 28 group-area combinations that had at least one measured non-roosting site and one measured roosting site that was actively used for roosting during the field survey. A few trees were represented multiple times in the dataset as they were used by different social groups at different periods.

#### 2.4.2. Do territorial borders and/or territorial conflicts affect roosting location?

**Figure 2.**
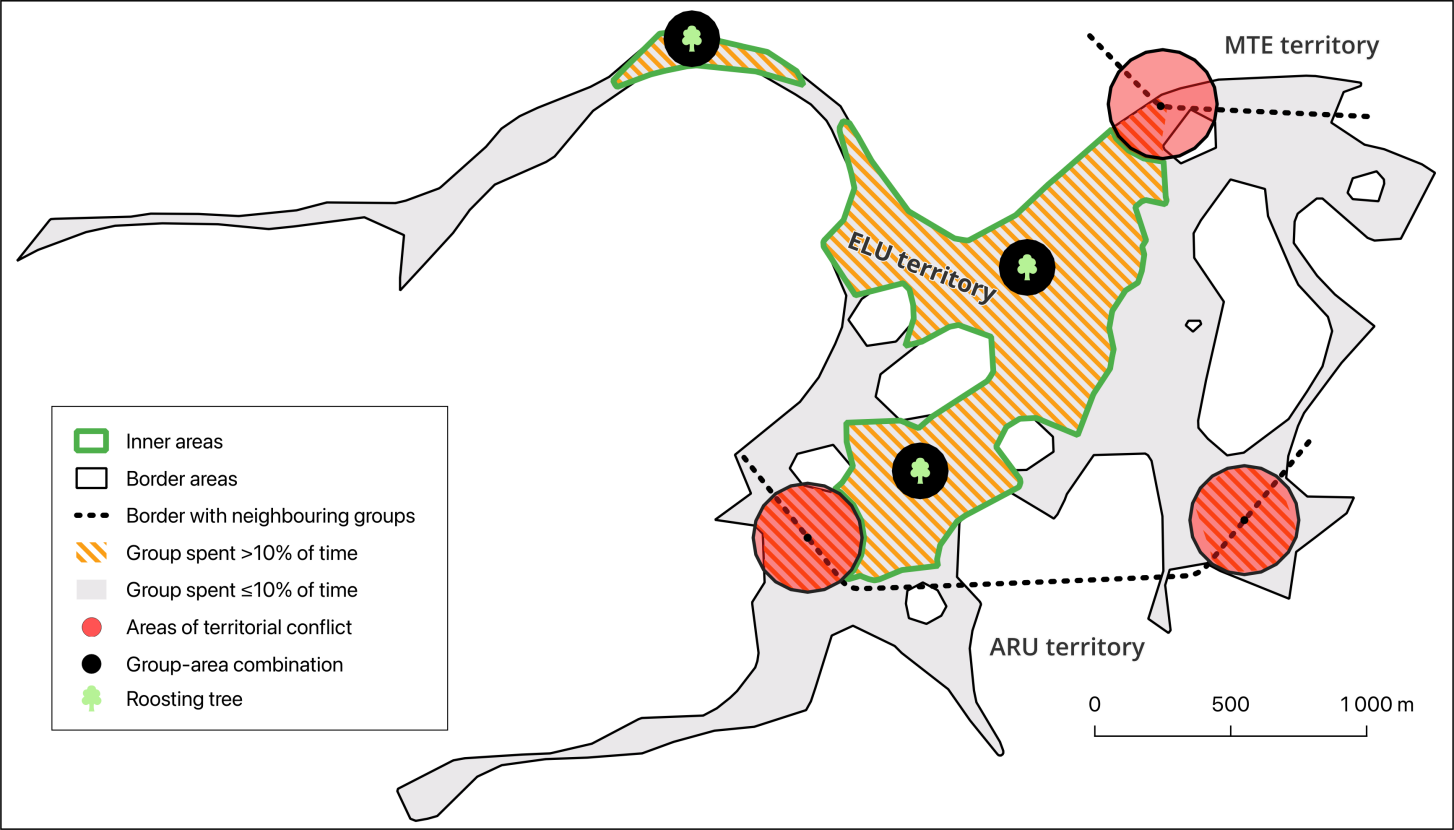
The territory of ELU group. Note the location of roosting trees in relation to the border (i.e. areas where the group spent ≤10% of time *or* within a 200 m radius from where territorial conflicts occurred) and inner areas (i.e. areas where the group spent >10% of time *and* were >200 m from where territorial conflicts occurred). The group always roosted in the inner parts of its territory despite participating in territorial conflicts on 6 out of 26 observation days.

Five social groups were extensively studied during morning (4 h) and/or evening (1–2 h) sessions across 186 days between November 2011 and July 2012. In each session, we recorded group movement, and for 108 of those dates, we also recorded whether a border encounter with another Arabian babbler group occurred. Group movement was recorded every 30 s with a GPS carried by the observer who followed the last group members, who usually stay within sight of other group members [13]. The home range of each group was calculated using the LoCoH software [47]. Areas where the group spent less than 5% of its time were excluded from the home range. Arabian babbler groups have distinct territorial borders with neighbouring groups that are easily identified during repeated confrontations that include calling and fighting within the same approximately 80 m area. Consequently, the home range was divided into border (i.e. areas where the group spent ≤10% of its time *or* within a 200 m radius from where territorial conflict occurred) and inner areas (i.e. areas where the group spent >10% of its time *and* were >200 m from where territorial conflict occurred; figure 2).

A GLMM was used to test whether encounters with non-group member conspecifics during the day affect roosting location during the following night. The response variable was the location of the roosting tree (inner/border area). The fixed effect predictor was whether the group encountered non-group member conspecifics during the morning and/or evening observation (yes/no). Group ID was set as a random effect. The sample size of the model was 108 nights and five groups.

#### 2.4.3. Do group size and/or roosting site size affect the probability of groups splitting to roost in two sites?

Since measurements of canopy width were not available for all sites, we ran two similar GLMMs. In both models, the response variable was whether the group roosted in one site or split to roost in two different sites (cases where only the female separated from the group to incubate eggs in the nesting tree were considered as if the group roosted in the same site), group ID was set as a random effect, and numerical predictors were centred by subtracting the mean of the predictor from each data point. The full model included all nights, and group size was set as a single fixed effect (319 = nights, 24 groups). The reduced model included only nights where the group roosted on trees with canopy width data, and the interaction between group size and canopy width (or the narrowest part of a reed cluster) was set as a fixed effect (*n* = 189 nights, 16 groups).

### 2.5. Roosting site fidelity

#### 2.5.1. Is there a shortage of suitable roosting sites?

We evaluated the number of sites suitable for roosting within each territory by summing the number of typical roosting sites across all group-area combinations of the focal group (i.e. live trees and reed clusters with a canopy density index equal to or higher than that of the actual roosting site in the group-area combination). Note that this is a minimum estimation since group-area combinations covered only a small portion of the group’s territory (figure 2).

#### 2.5.2. Do groups use specific roosting sites significantly over nearby alternatives?

The random likelihood of roosting in each site within a group-area combination was calculated by dividing the number of nights the group roosted in the area by the minimum number of potential roosting sites in this area. Owing to the small number of nights and potential sites per group area combination, 10 000 *p*-values were simulated using Monte Carlo simulations, and a separate chi-square test of goodness-of-fit was used for each of the 35 group-area combinations with five or more observation nights (out of a total of 53 group-area combinations with data).

## 3. Results

### 3.1. Sample size

Between 2011 and 2025, we documented the use of 82 different roosting sites by 29 social groups during 940 nights (nights in which the group split to roost on two sites were considered twice).

### 3.2 Method validation

### 3.2.1. Do trained human observers alter roosting site usage?

Arabian babbler groups rarely changed roosting site during the night-time and were not more likely to do so when observed by a researcher (2% out of 132 nights) than in his absence (0% out of 118 nights of AudioMoth recording; full-null model comparison: $\chi_{1}^{2}$ = 1.82, *p* = 0.18; electronic supplementary material, figure S2).

Groups were also not likely to change to other roosting sites than their main site at the time in the presence of a researcher (0% out of 42 nights) than in his absence (5% out of 121 nights of AudioMoth recording; full-null model comparison: $\chi_{1}^{2}$ = 3.56, *p* = 0.06; electronic supplementary material, figure S2).

### 3.3. Ecological and social characteristics of roosting behaviour

#### 3.3.1. What characteristics are important for roosting sites?

**Figure 3.**
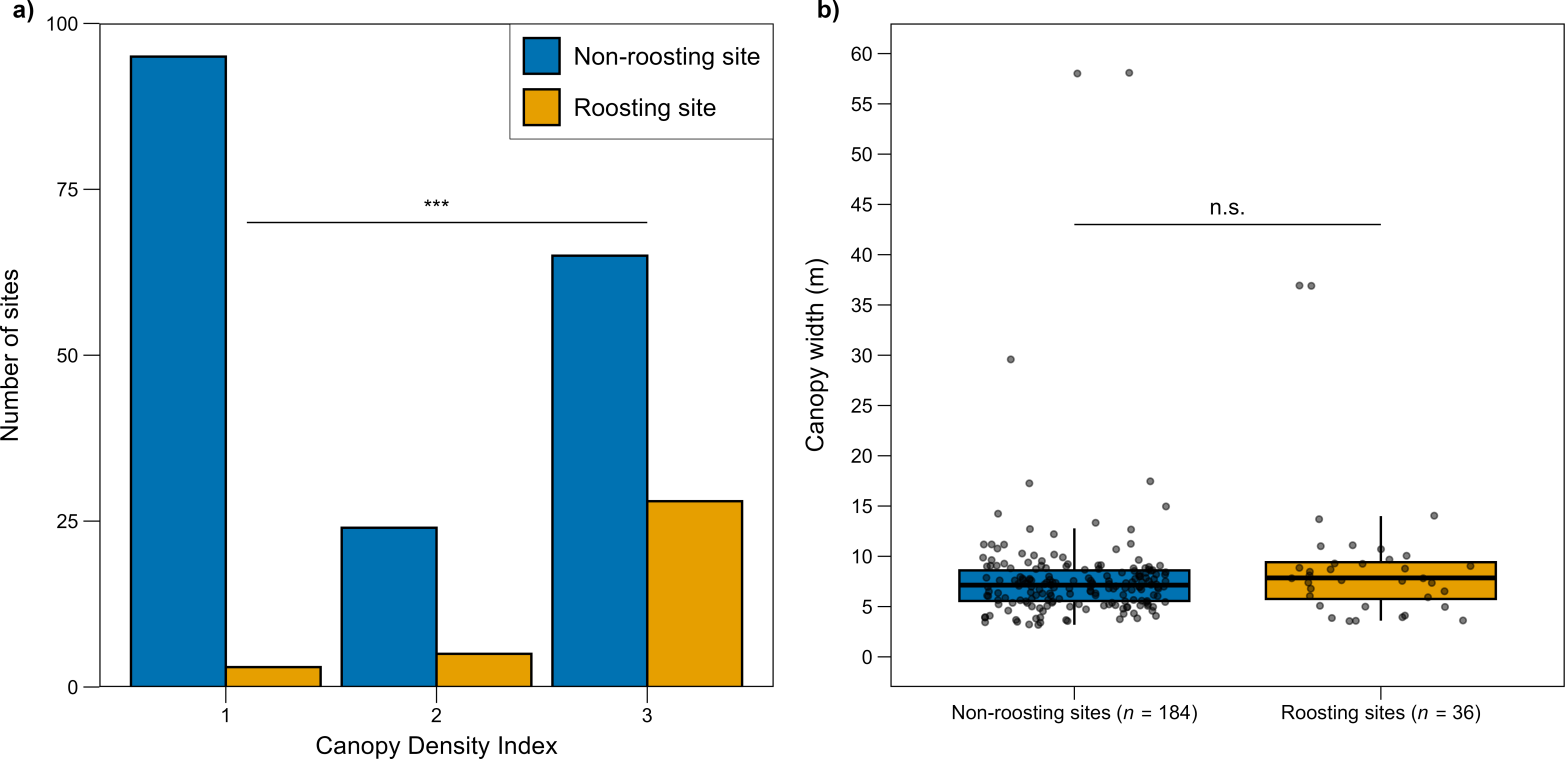
(a) Canopy density index score of non-roosting versus roosting sites. 1 = the main branches of the tree are clearly visible, 2 = the main branches of the tree are somehow visible, 3 = at least one main branch of the tree is hidden by dense foliage (e.g. figure 1b; electronic supplementary material, figure S1) and/or extensive growth of vines (e.g. *Commicarpus sinuatus* Meikle; figure 1a). (b) Canopy width of non-roosting versus roosting sites. Horizontal bars represent the median, boxes represent the 25 and 75% quartiles and points beyond the vertical lines are outliers. Raw data are presented as jittered points.

All roosting sites were live plants (*n* = 82; table 1). A roosting tree that died was abandoned (*n* = 1). We never observed Arabian babblers roost in inanimate objects (e.g. rock crevices or man-made structures). Canopy density had the most explanatory power for roosting site selection over its nearby alternatives (table 2 and figure 3). The size of reed clusters used for roosting varied between 0.0002 and 0.0185 km^2^ (median: 0.0055 km^2^, *n* = 6).

**Table 2.** Results of different generalized linear mixed models testing which characteristics affect the selection of roosting sites.

(a) Akaike Information Criterion (AIC) and Bayesian Information Criterion (BIC) scores of the five models with the lowest BIC					
fixed terms included in the model		AIC		BIC	ΔBIC
canopy index score		195.56		209.14	0
canopy index score + tree height		194.11		211.08	1.95
canopy index score + canopy width		195.25		212.21	3.07
canopy index score + tree height + canopy width		194.51		214.87	5.73
canopy index score + plant species		190.44		224.38	15.24

(b) best model					
**response variable:** the tree/reed cluster (was/was not) used for roosting **random effects:** group-area combination **full sample size:** 220 sites, 28 group-area combinations **full-null model comparison:** $\chi_{2}^{2}$= 34.22, *p* < 0.001					
term	estimate	s.e.	*χ* ^2^	d.f.	*p*
intercept	–5.89	0.63			
**canopy index score:**			**34.22**	**2**	**<0.001**
2	2.06	0.78			
3	2.98	0.65			

#### 3.3.2. Do territorial borders and/or territorial conflicts affect roosting location?

Ninety-five per cent of roosting sites were in the inner area of the group’s territory (*n* = 20 roosting sites, five groups; figure 2), and these inner sites were used on 97% of nights (*n* = 129 nights). Border encounters with conspecifics did not affect the probability of the group roosting at the inner (44 days with border encounters out of 106 roosting nights) or border areas of the territory during the following night (1 day with a border encounter out of two roosting nights; full-null model comparison: $\chi_{1}^{2}$ = 0.1, *p* = 0.75).

#### 3.3.3. Do group size and/or roosting site size affect the probability of groups splitting to roost in two sites?

**Figure 4.**
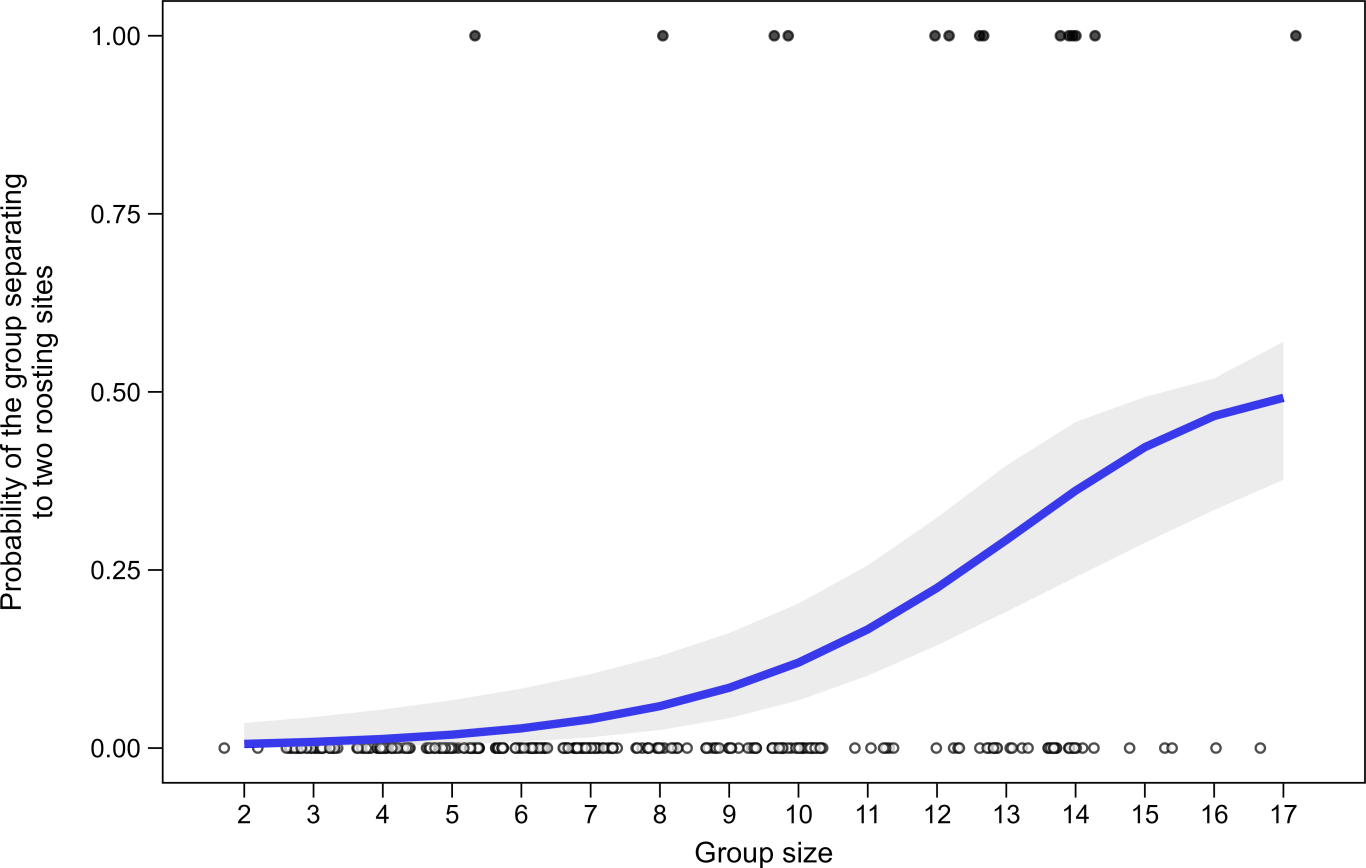
The probability of a social group splitting to roost in two sites as a function of group size (*n* = 319 nights, 24 groups).

Group size was positively associated with increased probability of splitting to roost in different sites (table 3a and figure 4). Group size also had a significant effect in the model with an interaction between group size and canopy width, despite reduced sample size (table 3b). Even when splitting, the separated parties of the same group roosted in nearby sites (mean ± s.e. distance: 78 ± 33 m, range: 25–204 m, *n* = 5 pairs of trees).

**Table 3.** Results of the group splitting generalized linear mixed models.

(a) response variable: the group separated to roost in two roosting sites (yes/no)					
**random effects:** tree ID. ***n* =** 319 nights, 24 groups **full-null model comparison:** $\chi_{1}^{2}$ = 11.91, *p* < 0.001					
term	estimate	s.e.	*χ* ^2^	d.f.	*p*
intercept	–6.90	1.11			
group size (centred)	0.40	0.09	11.91	1	0.001

**(b) response variable:** the group separated to roost in two roosting sites (yes/no)					
**random effects:** tree ID. ***n* =** 189 nights, 16 groups **full-null model comparison:** $\chi_{3}^{2}$ = 17.06, *p* = 0.001					
term	estimate	s.e.	*χ* ^2^	d.f.	*p*
intercept	–3.5319	0.5142			
group size (centred) * canopy width (centred)	–0.0005	0.0002	2.95	1	0.086
group size (centred)	0.2928	0.1038			
canopy width (centred)	–0.0011	0.0006			

### 3.4. Roosting site fidelity

#### 3.4.1. Is there a shortage of suitable roosting sites?

**Figure 5.**
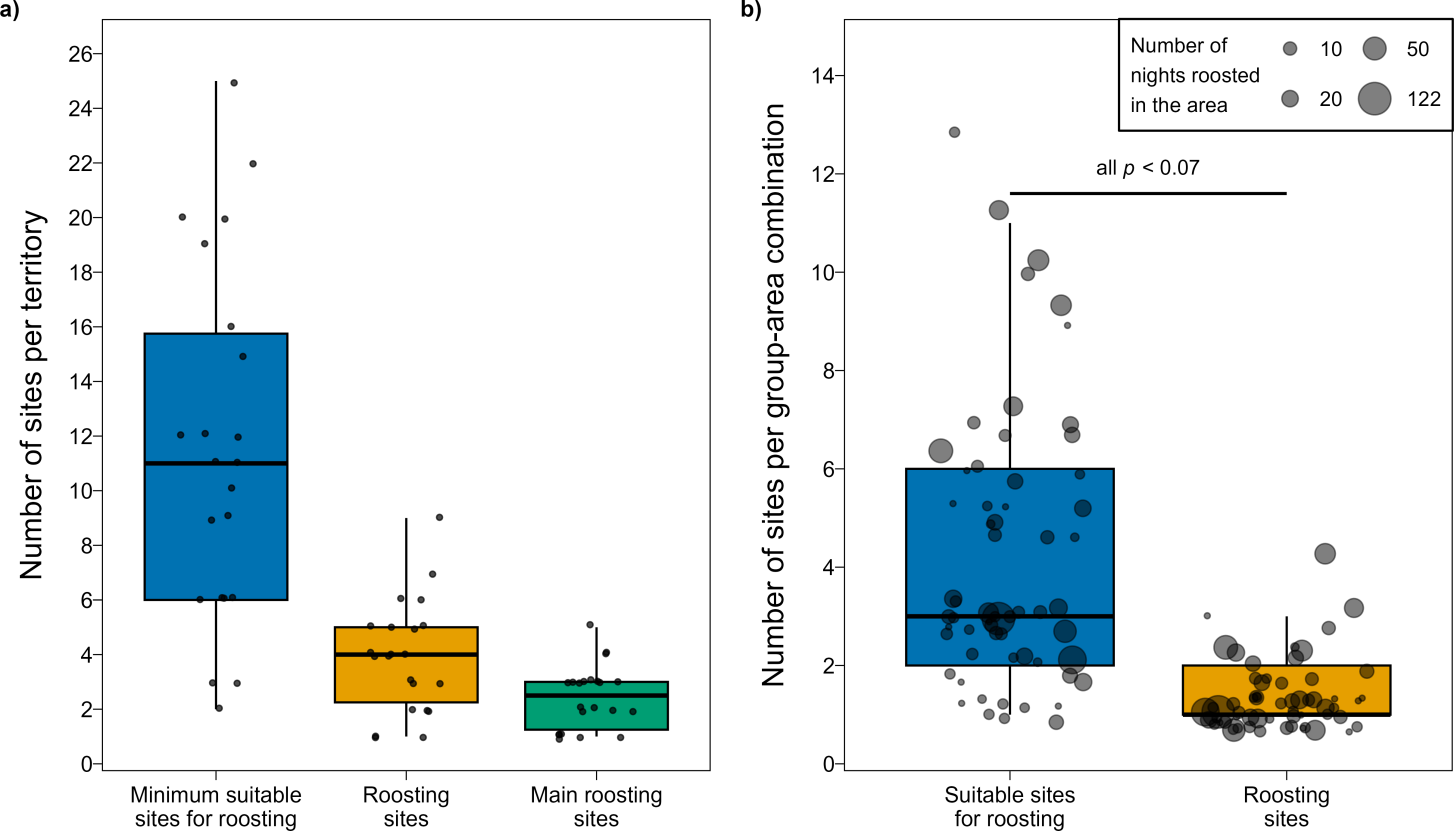
(a) The minimum number of suitable sites for roosting (i.e. a reed cluster or tree with canopy density index ≥ the actual roosting site), the number of sites that were used for roosting at least once and the number of sites used more than 10% of nights (i.e. main sites) in a territory (*n* = 22 territories). (b) The number of sites suitable for roosting within 100 m of an actual roosting site/s versus the number of sites used for roosting in this area (*n* = 61 group-area combinations). Horizontal bars represent the median, boxes represent the 25 and 75% quartiles and points beyond the vertical lines are outliers. Raw data are presented as jittered points.

Each of the 22 extensively studied groups had 11.6 ± 1.4 sites suitable for roosting within its territory (mean ± s.e.). Nonetheless, each group roosted in 3.9 ± 0.4 of these sites, of which only 2.4 ± 0.2 were ‘main’ sites that were used for more than 10% of nights (mean ± s.e.; figure 5a).

#### 3.4.2. Do groups use specific roosting sites significantly over nearby alternatives?

Although there were 4.2 ± 0.4 suitable sites within each group-area combination (*n* = 61), only 1.3 ± 0.1 of these sites were used for roosting (mean ± s.e.). Consequently, roosting sites were used at a non-random frequency over nearby alternatives (all *p* < 0.07, *n* = 35 group-area combinations with five or more observation nights; figure 5b; electronic supplementary material).

#### 3.4.3. How long does fidelity to roosting sites last?

Groups usually rotated between their main roosting sites every few weeks. For instance, during our across-nights experiment, six of the eight groups roosted in the same tree during the entire experiment (11–33 nights per group). The two other groups roosted in the same tree during 11 and 36 out of the 13 and 40 nights monitored, respectively.

Dynasties of two of the three social groups observed at the beginning of the study (i.e. 2011) still occupied the same territories at the end of the study (i.e. July 2025). These two dynasties were still using the same roosting trees (figure 1a; electronic supplementary material, figure S1) despite three and four complete changeovers in their group membership (electronic supplementary material, figure S3).

## 4. Discussion

### 4.1. Unbiased roosting research

Sleeping animals are vulnerable [5,48] and, therefore, sensitive to disturbance [49,50]. Disturbance during sleep may alter roosting behaviour in different ways, for instance, changing roosting site during the night-time [15,32], increasing roosting site changes across nights [6,7] and changing fidelity from a specific tree to a cluster of trees (if the animal avoids the exact site where it was disturbed but maintains fidelity to its familiar area). We propose that the presence of the above-described patterns in data from unhabituated animals should be confirmed not to be research artefacts—for example, by comparison with remote sensing monitoring as was done in this study (electronic supplementary material, figure S2)—before being accepted as natural roosting patterns [51].

### 4.2. Ecological characteristics of roosting sites

Several bird (white-winged chough *Corcorax melanorhamphos* [33], jungle babblers *Argya striata* [50]) and primate species (Geoffroy’s tamarin *Saguinus geoffroyi* [32], northern pygmy marmosets *Cebuella pygmaea* [34], reddish-grey mouse lemur *Microcebus griseorufus* [8]) were speculated to prefer dense canopy for roosting sites. To the best of our knowledge, we provide the first evidence for the necessity of a dense canopy in tree-roosting birds and mammals (table 2 and figure 3). In our study site, dense canopies were also created by extensive growth of vines on the tree [18,32]. The most common vine was *P. acacia*, whose evergreen, thick leaves [52] create closed structures (figure 1a). A dense canopy may provide a protective microclimate [8,10]. Indeed, Arabian babblers leave their roosting sites later after rainy nights [24], potentially because rain disturbs sleep [1]. Dense canopy may also protect from predators (e.g. owls [7,8]). This advantage is likely to apply to Arabian babblers (and other species) who usually avoid the outer parts of the canopy, making it difficult for nocturnal raptors to surprise them without making noise.

While canopy density was consistently included among the best models (table 2), it is reasonable to assume that canopy width and tree height also play some role in roosting site selection. First, our survey excluded most trees smaller than the actual roosting site in the group-area combination. Including these smaller trees may increase variation in the model to the point of a statistically significant effect for canopy width and/or tree height. Second, while our statistical approach selected the most parsimonious model (lowest BIC), alternative approaches (e.g. lowest Akaike Information Criterion) would have pointed out similar models that also include canopy width and/or tree height (table 2; [53]).

### 4.3. Sociality of roosting behaviour

Like in many cooperatively breeding birds [11,16,33,50] and primates [3,7,32,34], sleep is a social behaviour for Arabian babblers. Arabian babbler group members sleep in an intimate group huddle (figure 1c; [31]). Besides floaters and birds incubating in the nest [33], we never observed a group member roosting alone. Arabian babbler groups sometimes roost in sub-groups that clump along different branches of the same tree (always ≥2 animals), but seldom split to roost in different sites (figure 4a).

Roosting in large groups has the advantages of increased probability of at least one animal detecting a predator in advance (mallard, *Anas platyrhynchos* [5], gulls, *Larus* sp. [48]) and maintaining thermoregulation (chestnut-crowned babbler, *Pomatostomus ruficeps* [11]). Reducing group size by splitting to different sites thus requires explanation. The hypothesis that the roosting site is not large enough to accommodate all group members may hold for large primates (cotton-top tamarins [15]) or species roosting in small cavities (chestnut-crowned babbler [11], green woodhoopoe [12]). This explanation, however, may only explain a few cases where very large Arabian babbler groups had a roosting site with a relatively narrow canopy (table 3b), because all roosting sites had canopy widths many times larger (minimum canopy width: 3.6 m; figure 3) than the maximum group size observed in this study (i.e. 17 birds with a typical body width of approximately 5 cm each). An alternative explanation is that larger groups may include looser social relationships between main parties [17] and/or be more likely to include parties with different interests. This hypothesis is supported by the fact that most splitting events in our study occurred during relatively exceptional social events (e.g. the re-joining of a female to her old group, the death of a bird in the roosting tree, birds leaving the main roosting tree to sleep next to the nesting female).

Arabian babblers had most of their roosting trees away from their territory’s border (figure 2). Groups also did not tend to roost at the few roosting sites located in border areas following an encounter with conspecifics. These findings are in line with some species that usually roost at the inner areas of their territory (reddish-grey mouse lemurs [8], kinkajou *Potos flavus* [54]), but contrast with other species who often roost at their territory’s border, arguably to protect resources (e.g. fruit trees in Spix’s moustached tamarin [7] or roosting cavities in green woodhoopoe [12]). This difference may reflect the fact that Arabian babblers feed on more distributed prey, such as invertebrates [55] and/or their strong fidelity to a handful of roosting sites (figure 5).

### 4.4. Roosting site fidelity

We present, to our knowledge, the first systematic evidence supporting Zahavi’s [20] claim that Arabian babblers exhibit long-term fidelity to roosting sites. This fidelity can extend over at least four generations and 15 years (electronic supplementary material, figure S3). To the best of our knowledge, this is the longest roosting site fidelity shown for social groups of birds (e.g. jackdaw, *Corvus monedula* [22], white-winged chough [33]) and mammals (e.g. baboon, *Papio anubis* [6], chimpanzee, *Pan troglodytes verus* [56], cotton-top tamarins [15], Geoffroy’s tamarin [32], moustached tamarins, *Saguinus mystax* [7], reddish-grey mouse lemur [8], Honduran white bat, *Ectophylla alba* [10]), though the relatively short study periods of most of these studies may have hindered their ability to detect long-term fidelity.

Repeated usage of roosting sites may have disadvantages such as increased ectoparasite load in the site [57] and increased predictability for predators [8]. Strong fidelity is therefore often explained as the result of a shortage of alternative sites for roosting [8,16]. This hypothesis does not hold for Arabian babblers since suitable sites were abundant in their territories (figure 5). We suggest two alternative explanations for the strong fidelity exhibited by Arabian babblers. First, selected roosting sites may provide benefits that were not examined in this study. For example, thicker foliage may create a slightly superior microclimate against temperature and/or wind [8,11]. An additional explanation may be that groups develop a habit for specific sites and maintain it as a social tradition [58]. This hypothesis can be tested when a new group takes over the territory of another. If the new group uses the same roosting sites as the previous territory owners, that would suggest that roosting sites are selected because of merits that make them the best available options. By contrast, if the new group roosts in different sites than the previous territory owners, that would suggest a role of social traditions [58,59].

### 4.5. Conservation

Sleep in a familiar and protected site is important for animals’ health [1,6]. Our findings highlight the importance of specific roosting sites for Arabian babblers, specifically those large, dense acacia trees and reed clusters. Unfortunately, the number of these plants has decreased in the Great Rift Valley of Israel owing to agricultural expansion [60] and road construction [61]. Understanding the characteristics for which roosting sites are selected and mapping the geographical locations of frequently used roosting sites are therefore important to guide conservation in a world under increasing anthropogenic disturbance [18,22].

## Data Availability

The data used for this study, the code used for analyses and the results are provided as R data frames, R code and R objects in the electronic supplementary material. The geographical coordinates of the roosting sites were omitted to prevent disturbance to wildlife.
